# Gut metabolite Urolithin A mitigates ionizing radiation‐induced intestinal damage

**DOI:** 10.1111/jcmm.16951

**Published:** 2021-10-01

**Authors:** Yuanyang Zhang, Yinping Dong, Ping Lu, Xinyue Wang, Wenxuan Li, Hui Dong, Saijun Fan, Deguan Li

**Affiliations:** ^1^ Tianjin Key Laboratory of Radiation Medicine and Molecular Nuclear Medicine Institute of Radiation Medicine Chinese Academy of Medical Science & Peking Union Medical College Tianjin China

**Keywords:** apoptosis, gut microbes, ionizing radiation, radiation enteritis, UroA

## Abstract

Ionizing radiation (IR)‐induced intestinal damage is the major and common injury of patients receiving radiotherapy. Urolithin A (UroA) is a metabolite of the intestinal flora of ellagitannin, a compound found in fruits and nuts such as pomegranates, strawberries and walnuts. UroA shows the immunomodulatory and anti‐inflammatory capacity in various metabolic diseases. To evaluate the radioprotective effects, UroA(0.4, 2 and 10 mg/kg) were intraperitoneally injected to C57BL/6 male mice 48, 24, 1 h prior to and 24 h after 9.0Gy TBI. The results showed that UroA markedly upregulated the survival of irradiated mice, especially at concentration of 2 mg/kg. UroA improved the intestine morphology architecture and the regeneration ability of enterocytes in irradiated mice. Then, UroA significantly decreased the apoptosis of enterocytes induced by radiation. Additionally, 16S rRNA sequencing analysis showed the effect of UroA is associated with the recovery of the IR‐induced intestinal microbacteria profile changes in mice. Therefore, our results determinated UroA could be developed as a potential candidate for radiomitigators in radiotherapy and accidental nuclear exposure. And the beneficial functions of UroA might be associated with the inhibition of p53‐mediated apoptosis and remodelling of the gut microbes.

## 1 INTRODUCTION

Cancer has been the worldwide disease, expected to be above 23 million patients suffering from various types of cancer by 2030.^1^ Ionizing radiation(IR) is essential and effective in clinical diagnoses and cancer treatment. While intestinal damage is the major side effect for definitive radiotherapy of the abdominal cancer such as pancreatic, gastric and colorectal cancer. IR destroys the intestinal architecture and causes severe life‐threatening intestinal damage in clinic.^2^ Hence, it is crucial to find a way to attenuate the intestinal damage induced by radiotherapy treatment in clinic.

Ionizing radiation causes gut microbes disorders, raises the susceptibility of intestinal tract and participates in the pathogenesis of intestinal diseases.^3^ The gut microbes were pivotal in radiation enteritis induction, and faecal microbiota transplantation helped irradiated mice and radiotherapy patients recovery.^4^, ^5^ Further researches found that gut metabolites were critical in restoring the composition of gut microbes and improving radiation‐induced intestinal damage. These studies suggested that gut metabolites could be alleviating IR‐induced intestinal damage.

Urolithin A (UroA) is a gut metabolite of ellagitannin, a compound in fruits and nuts such as pomegranates, strawberries and walnuts. It shows various pharmacological effects, including regulating oestrogen receptor and androgen receptor, antioxidant, anti‐inflammation and anti‐ageing ability and so on. UroA can restore the colon damage induced by high‐fat diet and regulate the gut microbiota.^6^ But the effects of UroA in IR‐induced intestinal damage are still unknown.

In present research, we detected whether UroA ameliorates radiation‐induced intestinal damage, and further explored the possible mechanism involved. The results proved UroA markedly elevated the survival rate and the average survival days of mice exposed by lethal dose of IR and improved the intestinal tissue morphology architecture and the regeneration ability of enterocytes. Therefore, the inhibition of p53‐mediated apoptosis and the reshaping of gut microbes are indispensable for the beneficial effects of UroA. We demonstrated that UroA might be a potential candidate for radiomitigators in radiotherapy and accidental nuclear exposure.

## 2 MATERIALS AND METHODS

Detailed materials and methods are provided in the Appendix S1 section.

## 3 RESULTS AND DISCUSSION

### 3.1 UroA improves the survival and intestinal morphology and function of mice after 9.0 Gy IR

**FIGURE 1:**
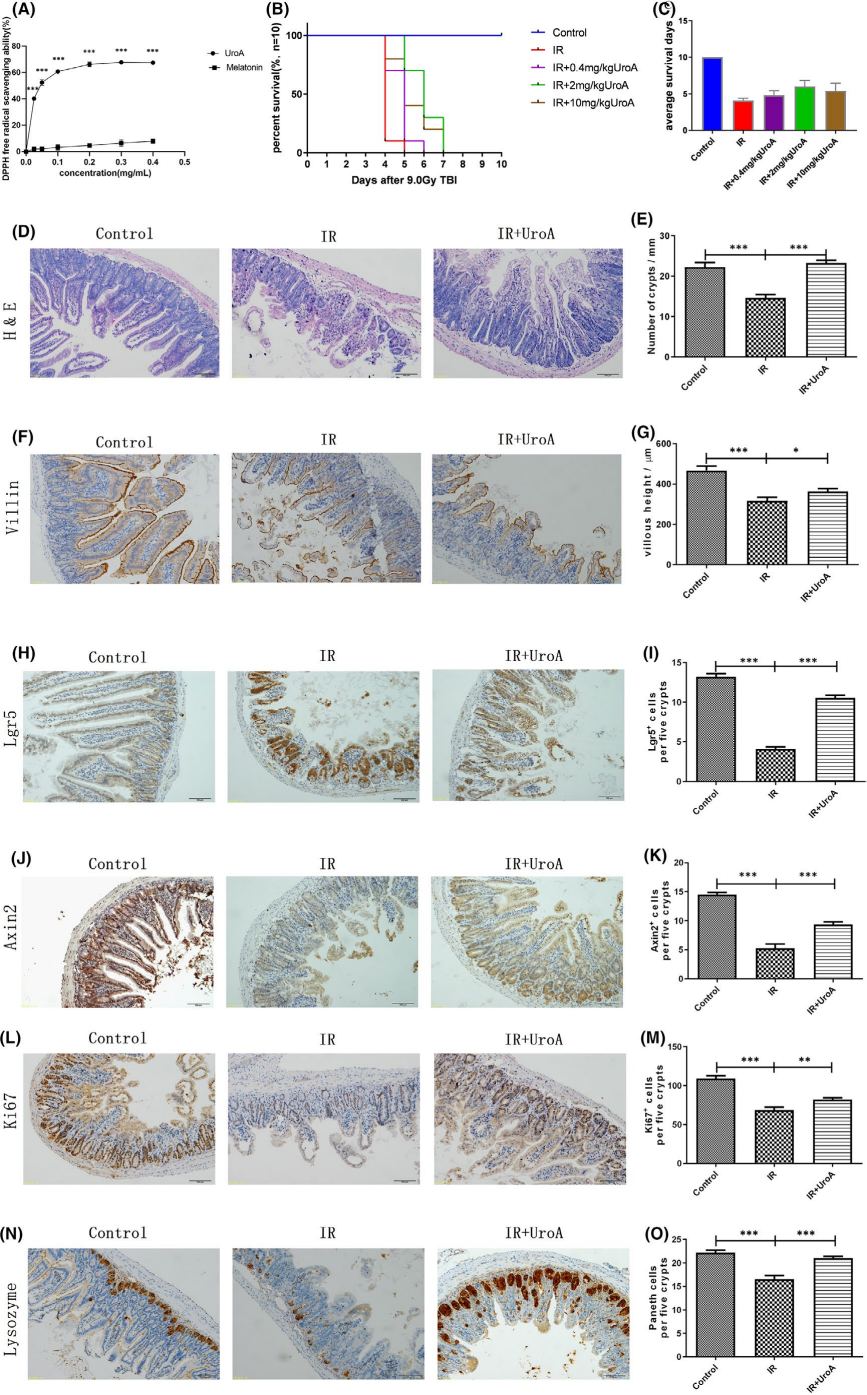
UroA shows excellent antioxidant capacity and improves the survival and intestinal morphology and function of mice after 9.0 Gy TBI. (A) The antioxidant capacity of UroA and melatonin was compared and detected by DPPH. The DPPH free radical scavenging activities by 0.025(*p *< 0.001, 95% confidence interval: −41.86 to −34.63), 0.05(*p *< 0.001, 95% confidence interval: −54.13 to −46.34), 0.1(*p *< 0.001, 95% confidence interval: −61.26 to −53.75), 0.2(*p *< 0.001, 95% confidence interval: −65 to −57.98), 0.3(*p *< 0.001, 95% confidence interval: −65.12 to −57.27), 0.4 (*p *< 0.001, 95% confidence interval: −62.83 to −56.09) mg/ml of UroA were significantly higher than that of melatonin. n = 3 samples per group. ∗∗∗*p* < 0.001. (B) The survival of mice in three groups. Log‐rank (Mantel‐Cox) test suggested that compared with the IR group, all three doses of UroA (0.4, 2 and 10 mg/kg) significantly developed the survival of mice exposed by 9.0 Gy TBI (*p *= 0.0076, *p *< 0.001 and *p *= 0.0015). (C) The average survival days of mice in five groups. One‐way ANOVA test suggested that compared with the control group, the average survival days of IR group had significantly reduced (*p *< 0.001, 95% confidence interval: −5.477 to −4.723); compared with the IR group, the average survival days of UroA (0.4, 2 and 10 mg/kg) group had significantly increased (*p *= 0.1649, 95% confidence interval: −1.566 to 0.166, *p *< 0.001, 95% confidence interval: −2.766 to −1.034 and *p *= 0.0009, 95% confidence interval: −2.166 to −0.434). The mice in the control group and the IR group were injected intraperitoneally with phosphate‐buffered saline, and the mice in the IR+UroA group were injected intraperitoneally with 0.4, 2 or 10 mg/kg UroA at 48, 24, 1 h before and 24 h after the 9.0 Gy TBI. n = 10 mice per group. **p < 0.01, ***p < 0.001. (D) Representative haematoxylin and eosin staining images of small intestine. (E) The quantified results of crypts counting. Two‐tailed unpaired t test suggested that compared with the control group, the number of crypts in IR group significantly reduced (***p < 0.001, 95% confidence interval: 4.746‒10.50); compared with the IR group, the numbers of crypts in UroA group significantly increased (***p < 0.001, 95% confidence interval: −10.87 to −6.435). (F) Representative Villin immunostaining images of the small intestinal tract. (G) The quantified results of villous height. Two‐tailed unpaired t test suggested that compared with the control group, the villous height in IR group significantly reduced (***p < 0.001, 95% confidence interval: 94.86‒204.9); compared with the IR group, the villous height in UroA group significantly increased (*p* = 0.0378, 95% confidence interval: −91.17 to −2.734). n = 5 mice per group. *p < 0.05, **p < 0.01, ***p < 0.001. (H) Representative photomicrographs of intestinal sections showing Lgr5+ positive immunostaining cells stained by IHC. (I) Quantification of Lgr5+ positive immunostaining cells in five intestinal crypts. Two‐tailed unpaired t test suggested that compared with the control group (13.2 ± 0.4), Lgr5+ cells in IR group (4.1 ± 0.3) significantly reduced (***p < 0.001, 95% confidence interval: −10.15 to −8.028); compared with the IR group, Lgr5+ cells in UroA group (10.6 ± 0.3) significantly increased (***p < 0.001, 95% confidence interval: 5.529 to 7.38). (J) Representative photomicrographs of intestinal sections showing Axin2+ positive immunostaining cells stained by IHC. (K) Quantification of Axin2+ immunostaining cells in five intestinal crypts. Two‐tailed unpaired t test suggested that compared with the control group (14.5 ± 0.4), Axin2+ cells in IR group (5.3 ± 0.7) significantly reduced (***p < 0.001, 95% confidence interval: −10.96 to −7.545); compared with the IR group, Axin2+ cells in UroA group (9.4 ± 0.5) significantly increased (***p < 0.001, 95% confidence interval: 2.289 to 5.938). (L) Representative photomicrographs of intestinal sections showing Ki67+ positive immunostaining cells stained by IHC. (M) Quantification of Ki67+ immunostaining cells in five intestinal crypts. Two‐tailed unpaired t test suggested that compared with the control group (109.2 ± 3.4), Ki67+ cells in IR group (68.4 ± 3.9) significantly reduced (***p < 0.001, 95% confidence interval: −52.76 to −28.84); compared with the IR group, Ki67+ cells in UroA group (82.1 ± 2.5) significantly increased (*p* = 0.0095, 95% confidence interval: 3.964 to 23.44). (N) Representative photomicrographs of intestinal sections showing Lysozyme positive immunostaining cells stained by IHC. (O) Quantification of Lysozyme positive immunostaining cells in five intestinal crypts. Two‐tailed unpaired t test suggested that compared with the control group (22.2 ± 0.5), paneth cells in IR group (16.5 ± 0.8) significantly reduced (***p < 0.001, 95% confidence interval: −7.612 to −3.668); compared with the IR group, paneth cells in UroA group (21.0 ± 0.4) significantly increased (***p < 0.001, 95% confidence interval: 2.637 to 6.31). Results are presented as mean ±SD of n = 5 mice per group. *p < 0.05, **p < 0.01, ***p < 0.001. scale bar: 100 μm

To determine the antioxidant activity of UroA, the DPPH radical scavenging assay was performed. We applied melatonin as the positive control for its antioxidant capacity of driving the synthesis of superoxide dismutase and glutathione peroxidase.^7^, ^8^ As Figure 1A shown, the DPPH free radicals scavenging ability of UroA significantly increased in a dose‐dependent manner between concentration range from 0.025 to 0.4 mg/ml, and it is much stronger than melatonin of the same concentration.

Three doses of UroA(0.4, 2 and 10 mg/kg) were intraperitoneally injected to mice 48, 24, 1 h prior to and 24 h after 9.0 Gy TBI, and monitored for 10 days. Compared with the IR group, all three doses of UroA could improve the survival rate of irradiated mice. But the 2 mg/kg UroA performed best (*p *< 0.001). We found that all mice were died in the IR group at 5 days after 9.0 Gy TBI (Figure 1B), 10% survival in 0.4 mg/kg UroA group, 70% survival in 2 mg/kg UroA group and 40% survival in 10 mg/kg UroA group. As shown in Figure 1C, the average survival days of mice are 4.1 days (the IR group), 4.8 days (the IR + 0.4 mg/kgUroA group), 6.0 days (the IR + 2 mg/kgUroA group) and 5.4 days (the IR + 10 mg/kgUroA group). Results above indicated that UroA could improve the survival of mice exposed with lethal dose IR, and 2mg/kg performed best. Therefore, we selected 2 mg/kg UroA as the dose of following intestinal analyses.

As shown in Figure 1D‐G, the images of haematoxylin and eosin staining showed the identical enterocytes death, the villus incompletion, crypts drop out and mucosal inflammation. At the 3rd day after 9.0 Gy TBI, the crypts numbers of the small intestine in IR group significantly decreased compared with the control group (*p *< 0.001). Compared with the mice in the IR group, IR + UroA group showed more survival crypts (*p *< 0.001). The villin height of the IR group was also markable lower compared with the control group, while the UroA treatment could rescue the loss of villin height reduced by 9.0 Gy TBI. Collectively, we suggested that UroA can protect against the intestinal tract morphology damage of mice induced by IR.

Failure to maintain normal mucosal structure and function after IR indicates the defection of regeneration ability. After IR 3 days, the number of Lgr5^+^ positive cells was notably decreased in the small intestine, while the loss of Lgr5^+^ was relieved by UroA (Figure 1H‐I). It was reported that the protein expression level of Axin2 (Axis inhibition protein 2), a downstream molecule of β‐catenin, is critical for the intestinal cell proliferation and differentiation.^9^ It reduced remarkably on 3 days after 9.0 Gy TBI. However, UroA could alleviate the loss of Axin2. The expression level of Ki67, was obviously lower in the IR group compared with the control group, while UroA treatment could significantly promote the crypts regeneration with more Ki67 expression marked in the IR+UroA group. Lysozyme as the paneth cells marker showed the similar pattern of expression change as the Ki67, UroA rescued the reduction of lysozyme induced by radiation. Paneth cells could cultivate and protect ISCs by producing lysozymes involved in forming inflammatory disease. Therefore, the improvement of small intestine integrity in IR+UroA group could associate with promoting differentiation and proliferation as well as enhancing regenerative response of ISCs.

### 3.2 UroA alleviates DNA injury and apoptosis level of mice after 9.0 Gy IR

**FIGURE 2:**
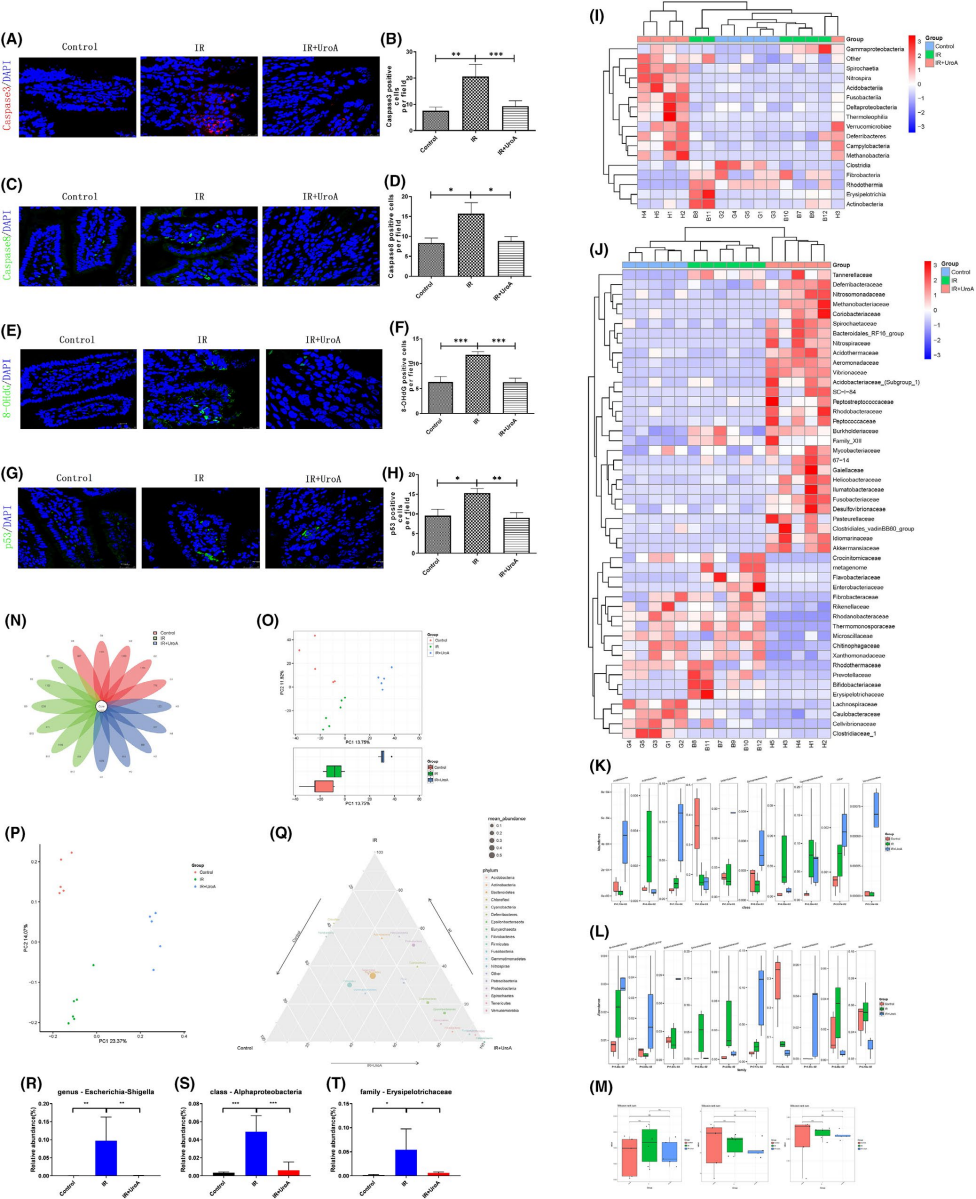
UroA alleviates DNA injury and apoptosis level and recovers the changes of gut microbiota in mice after 9.0 Gy TBI. (A) Representative photomicrographs of intestinal sections showing caspase3+ cells stained by IF (red,caspase3; blue, DAPI). (B) Quantification of caspase3+ cells. Two‐tailed unpaired t test suggested that compared with the control group (7.6 ± 1.4), caspase3+ cells in IR group (20.6 ± 4.6) significantly increased (*p* = 0.0097, 95% confidence interval: 3.682 to 22.35); compared with the IR group, caspase3+ cells in UroA group (9.3 ± 2.0) significantly decreased (*p* = 0.0303, 95% confidence interval: −21.24 to −1.232). (C) Representative photomicrographs of intestinal sections showing caspase8+ cells stained by IF (green,caspase8; blue,DAPI). (D) Quantification of caspase8+ cells. Two‐tailed unpaired t test suggested that compared with the control group (8.3 ± 1.2), caspase8+ cells in IR group (15.7 ± 2.8) significantly increased (*p* = 0.0299, 95% confidence interval: 0.8089 to 13.86); compared with the IR group, caspase8+ cells in UroA group (8.8 ± 1.1) significantly decreased (*p* = 0.0311, 95% confidence interval: −13.03 to −0.7015). (E) Representative photomicrographs of intestinal sections showing 8‐OHdG+cells stained by IF (green, ‐OHdG; blue,DAPI). (F) Quantification of 8‐OHdG +cells. Two‐tailed unpaired t test suggested that compared with the control group (6.3 ± 1.1), 8‐OHdG+cells in IR group (11.8 ± 0.7) significantly increased (*p* = 0.0008, 95% confidence interval: 2.767 to 8.066); compared with the IR group, 8‐OHdG+cells in UroA group (6.3 ± 0.8) significantly decreased (*p* = 0.0002, 95% confidence interval: −7.811 to −3.189). (G) Representative photomicrographs of intestinal sections showing p53+ cells stained by IF (green,p53; blue, DAPI). (H) Quantification of p53+ cells. Two‐tailed unpaired t test suggested that compared with the control group (9.6 ± 1.6), p53+ cells in IR group (15.3 ± 1.2) significantly increased (*p* = 0.0183, 95% confidence interval: 1.129 to 10.33); compared with the IR group, p53+ cells in UroA group (9.0 ± 1.3) significantly decreased (*p* = 0.0044, 95% confidence interval: −10.26 to −2.31). (I) The heatmap picture of gut microbes at class level calculated by ANOVA algorithm. (J) The relative abundance of gut microbes at the family level displayed in heatmap, which was calculated by ANOVA algorithm. (K) The abundance of top 10 different gut microbes at the class level. (L) Relative abundance of top 10 different gut microbes at family level. (M) The alpha diversity of the gut microbes. The Chao1 index of each group. The Shannon index of each group. The Simpson index of each group. (N) The flower plot of gut microbes. The number in core represents the common OTUs in all samples, and the number on the petal represents the total OTUs of each sample minus the number of common OTUs. (O) The PCA plot of gut microbes, in which PC1 is 13.75% and PC2 is 11.82%. The group IR+UroA has significant difference compared with the others. (P) The PCoA plot of gut microbes, in which PC1 is 23.37% and PC2 is 14.07%. (Q) The ternary figure at the phylum level. (R) UroA relieved the change of Escherichia shigella in genus level. One‐way ANOVA suggested that compared with the control group, Escherichia shigella in IR group had significantly increased (*p* = 0.0038, 95% confidence interval: −0.1593 to −0.03489); compared with the IR group, Escherichia shigella in UroA group had significantly decreased (*p* = 0.0039, 95% confidence interval: 0.03432 to 0.1587). (S) UroA relieved the change of Alphaproteobacteria in class level. One‐way ANOVA suggested that compared with the control group, Alphaproteobacteria in IR group had significantly increased (*p* = 0.0001, 95% confidence interval: −0.06552 to −0.0256); compared with the IR group, Alphaproteobacteria in UroA group had significantly decreased (*p* = 0.0004, 95% confidence interval: 0.0217 to 0.06426). (T) UroA relieved the change of Erysipelotrichaceae in family level. One‐way ANOVA suggested that compared with the control group, Erysipelotrichaceae in IR group had significantly increased (*p* = 0.0130, 95% confidence interval: −0.09392 to −0.01189); compared with the IR group, Erysipelotrichaceae in UroA group had significantly decreased (*p* = 0.0209, 95% confidence interval: 0.007713 to 0.08974). Results are presented as mean ±SD of n = 5 mice per group. *p < 0.05, **p < 0.01, ***p < 0.001. scale bar: 25 μm

To determine the DNA oxidative damage induced by ROS, 8‐Hydroxydeoxyguanosine (8‐OHdG) indicating DNA damage was investigated by IF staining. As shown in Figure 2E‐F, IR could significantly increase the 8‐OHdG content in the small intestine, and UroA could significantly alleviate the increase in 8‐OHdG in small intestine of mice exposed with radiation.

Since p53 protein is the key molecule responding to the cellular DNA injury by triggering apoptosis or inducing a cell cycle arrest, we assessed the level of p53 protein by IF analysis. As shown in Figure 2G‐H, IR induced the overexpression of p53, while the p53 in intestinal tissue was remarkably restored to the normal level by the administration of UroA. As shown in Figure 2A‐D, the upregulation of caspase8 and caspase3 was observed in the small intestine of mice exposed with 9.0 Gy TBI, while the effect was distinctly prevented by the treatment of UroA. These results indicated that UroA could downregulate the p53‐mediated apoptosis pathway downstream molecules to ameliorate the radiation‐induced intestinal damage.

### 3.3 UroA recovers the changes of gut microbiota in mice after TBI

Gut microbes play a crucial role in gastrointestinal pathology caused by IR, and UroA was studied previously to modulate the profile of gut microbes. Therefore, the effect of UroA in the gut of irradiated mice might be associated with the composition change of the gut microbes. Faecal samples of irradiated mice were harvested 3 days after IR, and 16S ribosomal RNA analyses of microbiota were performed. As shown in the flower plot (Figure 2N), among the 4772 identified operational taxonomic units (OTUs), 107 of them were the core OTUs that exist in each sample. As shown in Figure 2Q, the microbes *Euryarchaeota* and *Nitrospirae* are rich in the IR + UroA group and lack in the IR group, which associate with the benefit mechanism of UroA on radioprotective property. The main composition of phylum *Euryarchaeota* is *Methanogens* which is flourish in inflammatory bowel disease patient compared with healthy individual.^10^ The loss of *Nitrospirae* and the flourish of *Actinobacteria* are two signs associated with histological stages of gastric carcinogenesis.^11^ In our study, IR obviously upregulated the abundance of *Actinobacteria* compared with control group; and UroA relieved this change of community structure. To determine the alpha diversity of the gut microbes, the Chao1 index, the Shannon index and the Simpson index (Figure 2M) using pairwise comparisons using Wilcoxon rank‐sum test were used. There was no significant difference among three groups, suggested species diversity of microbial composition between each group is limited. As for beta‐diversity, the principal component analysis (PCA) (Figure 2O) and the principal co‐ordinates analysis (PCoA) (Figure 2P) revealed that irradiation caused an obvious change in the structure of gut microbes compared with the control group, while UroA treatment was confirmed a distinct segregation in the gut microbes profile paralleled with the IR group.

In order to clarify the changes of IR and UroA on gut microbe composition of irradiated mice, we performed statistical analyses at levels of the class and family, and drew heatmaps based on the relative abundance of different species. As seen in Figure 2I, the results did not show obviously differences among three groups at classes level. Contrarily, As shown in Figure 2J, 9 Gy IR exposure increased the relative abundance of the family *Flavobacteriaceae*, *Enterobacteriaceae*, *Bifidobacteriaceae and Erysipelotrichaceae*. However, UroA treatment ameliorated the bacteria differences induced by 9.0 Gy IR. Relative abundance of top 10 different gut microbes at the class and family level showed in Figure 2K‐L, and the abundance of *Enterobacteriaceae* and *Erysipelotrichaceae* in IR group was upregulated compared with others (*p *< 0.05).

Recently, the association between gut microbes and host health has been one of the most popular research topics. Studies have found that microbial metabolites promote the proliferation and regeneration of ISCs.^12^ Owing to the effect of anti‐inflammation property in protecting against radiation‐induced intestinal damage, we wonder whether UroA to some extent benefits organism by the influence on gut microbe composition. To scrutinize the effect of UroA on intestinal tract gut microbe composition, our study presents 16S rRNA sequencing analyses for three groups. The results showed that IR could cause gut microbes imbalance and a downregulation of beta‐diversity, accordance to the clinical counterpart.^13^ However, the administration of UroA could significantly alleviate the disturbance of gut microbes and the decrease of gut microbes abundance caused by radiation damage, indicated that reshape of gut microbes is also a mechanism of UroA ameliorate radiation‐induced intestinal damage. *Escherichia shigella* could mediate the transfer of virulence protein into host cytoplasm, destroy epithelial integrity and functions and subsequently promote cell death.^14^ Our result showed that radiation flourished the abundance of *Escherichia shigella*, and UroA administration produced a significantly remission (Figure 2R). Since *Proteobacteria* plays an important role in facilitating inflammatory response by regulating IL‐10/IL‐17 balance and the production of TGF‐β, the upregulation of *Proteobacteria* level in gut reflects dysbiosis or a tender status of gut microbial community.^15^ In present study, we found that radiation exposure leaded the bloom of *Alphaproteobacteria*, and UroA administration sharply relieved it (Figure 2S‐T). *Erysipelotrichaceae* is associated with the host lipid metabolism, positively correlated with TNF‐α and cause inflammation in gut. We found that irradiation remarkedly increased the abundance of *Erysipelotrichaceae*, however, UroA administration slightly ameliorated this change. According to results above, we suggested the radioprotective capacity of UroA is associated with the remodelling of gut microbes.

In summary, we demonstrated that UroA improves the maintenance of the intestinal homeostasis and the ability of regeneration exposed with radiation. These benefits of UroA might be associated with the inhibition of p53‐mediated apoptosis and remodelling of the gut microbes. Therefore, UroA could be a potential target for radiomitigators in radiotherapy and accidental nuclear exposure.

## CONFLICT OF INTEREST

The authors declare that there is no conflict of interest.

## AUTHOR CONTRIBUTIONS

**Yuanyang Zhang:** Data curation (equal); Formal analysis (equal); Investigation (lead); Project administration (lead); Resources (equal); Writing‐original draft (lead). **Yinping dong:** Methodology (equal); Supervision (equal); Visualization (equal). **Ping Lu:** Methodology (equal); Supervision (equal); Visualization (equal). **Xinyue Wang:** Data curation (equal); Investigation (supporting); Project administration (supporting); Resources (equal); Writing‐original draft (supporting). **Wenxuan Li:** Data curation (equal); Investigation (supporting); Project administration (supporting); Resources (equal); Writing‐original draft (supporting). **Hui Dong:** Funding acquisition (supporting); Writing‐review & editing (equal). **Saijun Fan:** Conceptualization (equal). **Deguan Li:** Conceptualization (equal); Funding acquisition (equal); Writing‐review & editing (equal).

## Supporting information

Appendix S1

## Data Availability

The data used to support the findings of this study are available from the corresponding author upon request.
