# Supplementary material for: Gut metabolite Urolithin A mitigates ionizing radiation‐induced intestinal damage
Source: J Cell Mol Med. 2021 Oct 1;25(21):10306–12. doi: 10.1111/jcmm.16951 (PMC8572803; doi:10.1111/jcmm.16951)
Supplement: Supplementary file 1 — Appendix S1 [file JCMM-25-10306-s001.docx]

**Appendix S1**

**Supplementary information for**

Gut metabolite Urolithin A mitigates ionizing radiation-induced intestinal damage

Yuanyang Zhang, Yinping Dong, Ping Lu, Xinyue Wang, Wenxuan Li, Hui Dong, Saijun Fan, Deguan Li^,*^

Tianjin Key Laboratory of Radiation Medicine and Molecular Nuclear Medicine, Institute of Radiation Medicine, Chinese Academy of Medical Science & Peking Union Medical College, Tianjin 300192, China.

^*^Corresponding author. Institute of Radiation Medicine, Chinese Academy of Medical Science & Peking Union Medical Collage, No. 238, Baidi Road, Nankai district, Tianjin, 300192, China.

E-mail address: lideguan@irm-cams.ac.cn

2 MATERIALS AND METHODS

2.1 Reagents

UroA (CAS 1143-70-0) was purchased from Hefei Hirisun Phamatech Co. Ltd (Hefei, China). DPPH (CAS 1898-66-4) was obtained from Tokyo Chemical Industry. Melatonin (CAS 73-31-4) was purchased from Tokyo Chemical Industry.

2.2 DPPH assay

The antioxidant ability of UroA was determined by DPPH radical scavenging assay. The UroA and melatonin were dissolved separately in the absolute ethanol in the concentration of 0.025, 0.05, 0.10, 0.20, 0.30 and 0.40 mg/mL. The blank sample was filled with the absolute ethanol in the same volume. The absorbance of samples at 517 nm were tested after incubated at dark for 30 minutes. The DPPH free radical scavenging ability (%) = 100 × [(absorbance of blank - absorbance of sample)/absorbance of blank].

2.3 Mice maintenance and ethics approval

C57BL/6 male mice weighing 19-24 g were provided from Beijing HFK Bioscience. Mice were bred in daily changed cages at suitable temperature with free access of standard laboratory water and food.

The animal experiments were conducted under the NIH Animal Care and Use Guidelines with the permission of Animal Care and Ethics Committee of IRM-PUMC (IRM-dwll-2018062).

2.4 IR and Treatment

IR was performed by a Cs^137^ γ-radiation source (Atomic Energy of Canada Ltd) at a dose rate of 1.02 Gy/min.

For the survival experiment, mice were randomly distributed into 5 groups(n=10): the control group, the IR group, the IR+0.4mg/kgUroA group, the IR+2mg/kgUroA group and the IR+10mg/kgUroA group. UroA(0.4, 2 and 10mg/kg) were intraperitoneally injected to mice 48h, 24h, 1h prior to and 24h after 9.0Gy TBI. The control group and the IR group were administrated with phosphate-buffered saline. The IR group, the IR+0.4mg/kgUroA group, the IR+2mg/kgUroA group and the IR+10mg/kgUroA group were exposed with 9.0Gy TBI, and the mice were monitored daily for 10 days or all the irradiated mice died.

In other experiments, mice were randomized into three groups(n=5): the control group, the IR group and the IR+UroA group. The mice in IR+UroA group were treated with UroA(2mg/kg) by intraperitoneal injection 48h, 24h, 1h prior to and 24h after IR. The control group and the IR group were treated with phosphate-buffered saline. The IR and IR+UroA groups were exposed with 9.0Gy TBI. After 3 days of IR exposure, mice were sacrificed to harvest the small intestine or the feces for testing.

2.5 Histopathological Analysis

The small intestines were gathered and fixed after the mice were sacrificed. After sinking in the hematoxylin-eosin dye solution for 5 minutes, slides were detected by optical microscope. The number of intestinal crypts and the length of villin were counted by using ImageJ 1.37 software.

2.6 Immunohistochemistry Analysis

The three μm-thick sections were deparaffinized by heating at 56°C and immersing in graded ethanol and water. Then, in order to performing antigen retrieval, the slides were blocked using boiled 0.01mol/L citrate buffer. After blocked the nonspecific antigen-binding sites in BSA diluted in PBS for 1 hour, the slides were immersed with anti-villin(Abcam, 1:800), anti-Lgr5 antibody (Abcam, 1:50), anti-Axin2 antibody(Abcam, 1:200), anti-Ki67 antibody (Abcam, 1:300) or anti-lysozyme(Abcam, 1:800) for one night. Subsequently, the sections were immersed with the homologous secondary antibody and counterstained with hematoxylin. Finally, the positive cells were stained by diaminobenzidine (DAB) kit (Sigma) for 10 min. The images were collected by digital imaging microscope OLYMPUS DP72.

2.7 Immunofluorescence Analysis

Samples were harvested and stained as described above. Immunofluorescence was performed using following antibodies: anti-8OHdG (CST, 1:100), anti-p53(Ruiying Bio, 1:1000), anti-caspase3(CST, 1:100) and anti-caspase8(CST, 1:100). After incubated in secondary antibody for 1 hour, the slides were counterstained with DAPI. The images for immunofluorescence were collected by using confocal laser scanning microscope (LEICA MICROSYSTEMS CMS GMBH).

2.8 Gut microbiota analysis

Fecal samples were collected from rectum of mice. Genomic DNA was extracted from fecal samples using DNeasy Power Soil Kit(QIAGEN, USA). The quality and quantity of genome DNA were evaluated by Nanodrop machine and stored at -20℃ until use. After DNA extraction, PCR amplification of the 16S rRNA gene was carried out by using the Illumina MiSeq PE300 system. Low quality and ambiguous sequences in raw data were cut out using Trimmomatic software. The sequences were assembled by utilizing FLASH software and reduced noise by QIIME software. The functional differences among all samples were calculated by Kruskal-Wallis test.

2.9 Statistical Analysis

The results were expressed as the mean±SD, and the data were analyzed by using a one-way analysis of variance (ANOVA). In the event that ANOVA justified post hoc comparisons between experimental group means, these were conducted using the Tukey’s multiple paired comparison test. Differences were considered significant at p < 0.05. All analyses were performed using GraphPad Prism Software (San Diego, CA, USA).
